# Human Immunodeficiency Virus-Associated Chronic Lung Disease in Children and Adolescents in Zimbabwe: Chest Radiographic and High-Resolution Computed Tomographic Findings

**DOI:** 10.1093/cid/cix778

**Published:** 2017-09-04

**Authors:** Sujal R Desai, Arjun Nair, Jamie Rylance, Hilda Mujuru, Kusum Nathoo, Grace McHugh, Edith Majonga, John Metcalfe, Katharina Kranzer, Rashida A Ferrand

**Affiliations:** 1 Department of Radiology, The Royal Brompton and Harefield NHS Foundation, London; 2 Department of Radiology, Guy’s and St Thomas’ NHS Foundation Trust, London; 3 Liverpool School of Tropical Medicine, Pembroke Place, United Kingdom; 4 Department of Pediatrics and Child Health, College of Health Sciences, University of Zimbabwe, Avondale; 5 Biomedical Research and Training Institute, Harare, Zimbabwe; 6 London School of Hygiene and Tropical Medicine, United Kingdom; 7 Division of Pulmonary and Critical Care Medicine, University of California, San Francisco

**Keywords:** Sub-Saharan Africa, HIV, chronic lung disease, HRCT, chest X-ray

## Abstract

**Background:**

Chronic respiratory symptoms are common among children living with human immunodeficiency virus (HIV). We investigated the radiological features of chronic lung disease in children aged 6–16 years receiving antiretroviral therapy for ≥6 months in Harare, Zimbabwe.

**Methods:**

Consecutive participants from a HIV clinic underwent clinical assessment and chest radiography. Participants with an abnormal chest radiograph (assessed by a clinician) and/or those meeting a clinical case definition for chronic lung disease underwent high-resolution computed tomography (HRCT). Radiological studies were scored independently and blindly by 2 thoracic radiologists. Relationships between radiological abnormalities and lung function were examined.

**Results:**

Among 193 participants (46% female; median age, 11.2 years; interquartile range, 9.0–12.8 years), the median CD4 cell count was 720/µL (473–947/µL), and 79% had a human immunodeficiency virus (HIV) load of <400 copies/mL. The most common chest radiographic finding was ring/tramline opacities (55 of 193 participants; 29%). HRCT scans were evaluated in 84 participants (69%); decreased attenuation (present in 43%) was the dominant abnormality seen. The extent of decreased attenuation was strongly correlated with both the severity and extent of bronchiectasis (*r*_s_ = 0.68 and *P* < .001 for both). The extent of decreased attenuation was also negatively correlated with forced expiratory volume in first second of expiration (*r*_s_ = –0.52), forced vital capacity (*r*_s_ = –0.42), and forced expiratory flow, midexpiratory phase (*r*_s_ = –0.42) (*P* < .001 for all).

**Conclusions:**

The HRCT findings strongly suggest that obliterative bronchiolitis may be the major cause of chronic lung disease in our cohort. Further studies to understand the pathogenesis and natural history are urgently needed.

## Teaser

There is a substantial burden of chronic lung disease among treated children with human immunodeficiency virus infection in this study from Zimbabwe. The radiological findings, coupled with functional test results, indicate that the key pathological lesion is constrictive obliterative bronchiolitis.

The global scale-up of antiretroviral therapy (ART) has resulted in a substantial decline in the incidence of perinatally acquired infections, and in a dramatic improvement in survival of children with human immunodeficiency virus (HIV) infection [1]. Hence, children with HIV who would otherwise have died in infancy, are now reaching adolescence in large numbers, and the pediatric HIV epidemic has shifted from one characterized by very high early childhood mortality rates to one that is a chronic, treatable condition among older children and adolescents [2].

Cotrimoxazole prophylaxis and ART substantially reduce the risk of acute respiratory tract infections and tuberculosis [3, 4]. However, recent studies from sub-Saharan Africa have reported a high prevalence of chronic lung disease, even among older children receiving ART [5–7]. The typical clinical picture is of a chronic, usually nonproductive, cough, hypoxia, tachypnea, reduced exercise tolerance and decreased lung function, and “nonspecific” chest radiographic abnormalities [8]. Plain chest radiography, despite its ready availability, is insensitive, and terminology used to define abnormalities often inconsistent. The limited availability of diagnostic modalities in resource-limited settings hinders definitive diagnosis and, in the absence of an alternative diagnoses, presumptive tuberculosis treatment is often started for those with chronic respiratory symptoms [9].

We recently conducted a prospective study to investigate the prevalence of chronic respiratory disease in HIV-infected children and adolescents attending for routine HIV care who were well established on ART and receiving cotrimoxazole prophylaxis. Among these children, 25% had chronic respiratory symptoms and reduced lung function [10]. In the present study, we report the spectrum of abnormalities on high-resolution computed tomography (HRCT) among study participants and association of radiological abnormalities with lung function.

## METHODS

### Study Participants

HIV-infected children aged 6–16 years receiving routine outpatient HIV care at the Harare Central Hospital, the largest public-sector hospital in Harare, Zimbabwe, were consecutively recruited, with enrollment limited to the first 5 eligible children every weekday for logistical ease. Recruitment was limited to those receiving ART (first or second line) for ≥6 months. The 6-month cutoff for ART duration was selected to allow enough time for participants to achieve viral suppression and for immune reconstitution inflammatory syndrome to manifest, which usually occurs in the first few months after ART initiation. Children with tuberculosis (excluded through sputum smear and culture examination) or presenting with symptoms and signs of acute respiratory tract infection were excluded.

The study was approved by the Medical Research Council of Zimbabwe, the Harare Hospital Ethics Committee, the Biomedical Research and Training Institute Institutional Review Board, and the London School of Hygiene and Tropical Medicine Ethics Committee. Written informed consent from guardians and assent from children was obtained before enrollment.

### Data Collection

Study procedures are described in detail elsewhere [10]. Briefly, sociodemographic and clinical data including HIV treatment history, past illnesses, and current symptoms were collected through a nurse-administered questionnaire. Participants underwent a standardized clinical examination, including pulse oximetry, shuttle walk testing, and spirometry. Spirometry was performed using EasyOne World spirometers (ndd Medical Technologies), following American Thoracic Society standard procedures [11]. Up to 8 forced exhalations were recorded with patients sitting, and the only data analyzed were from individuals who produced 3 consistent traces that met American Thoracic Society quality criteria. The highest forced expiratory volume in first second of expiration (FEV_1_) and forced vital capacity measurements were used, with other indices recorded from the best trace (largest total of FEV_1_ and forced vital capacity). Normal spirometric ranges were defined using the GLI 2012 equation, which gives reference values specific for race and sex and takes into account height and age [12]. The lower limit of normal was defined as 1.64 standard deviations (SDs) below the mean expected value (which describes the lowest 10 centiles of the reference population as “abnormal”). CD4 cell counts were determined using the PIMA platform (Alere), and HIV load by means of the COBAS Ampliprep/Taqman 48 analyzer (Roche).

Study participants underwent standard posteroanterior plain chest radiography. Participants referred for HRCT of the chest included those with an abnormal chest radiograph (assessed initially by a respiratory physician, blinded to clinical data) and those meeting the clinical case definition of chronic lung disease (ie, the presence of ≥1 of the following: chronic cough for ≥1 month with no microbiological evidence of tuberculosis; oxygen saturation of <92% at rest or desaturation of ≥5% from baseline on exercise testing; Medical Research Council (MRC) dyspnea score of >1; resting tachypnea [respiratory >30/min for 6–12-year-olds or >24/min for 13–16-year-olds); and abnormal results of spirometry, as defined above.

Supine HRCT images (1-mm collimation with 10-mm interspacing) were acquired at full inspiration and reconstructed using a high-spatial-frequency lung algorithm with window settings appropriate for viewing the lung parenchyma (window centre, −550 HU; window width, 1500 HU). HRCT studies were anonymized and downloaded onto digital storage disks for subsequent review on high-resolution screens using an Osirix HD viewing platform (OsiriX, version 6.5.2; OsiriX Foundation). The chest radiographic and HRCT studies were scored independently and in separate sessions by 2 thoracic radiologists blinded to clinical data (S. R. D. and A. N., with 20 and 11 years’ experience respectively in the interpretation of thoracic imaging).

### Chest Radiographic and HRCT Scoring

Principal chest radiographic and HRCT patterns were defined according to the Fleischner Society Glossary of terms [13]. Chest radiographic abnormalities were quantified using a previously validated scoring system with low interobserver variability [14]. In brief, the overall extent of abnormal lung (irrespective of pattern) was graded semiquantitatively on a 5-point scale (0, normal lung; 1–4, 1%–25%, 26%–50%, 51%–75%, and 76%–100% abnormal lung, respectively). The proportional extents of the following principal radiographic patterns were then quantified: consolidation; reticular pattern; noncavitating nodules; cavitating nodules; ring and/or tramline opacities (reflecting the presence of thick-walled and/or dilated bronchi seen either “end-on” [rings] or in plane [tramlines]; ground-glass opacification; and masses. Nonprincipal patterns—namely, paucity of vessels, hilar and/or mediastinal lymph node enlargement, and hyperexpansion—were recorded as absent or present. The severity of volume loss, if present, was graded semiquantitatively (0, no volume loss; 1, mild; 2, severe).

HRCT patterns were quantified visually in 6 lobes (note that the lingula was considered as a separate lobe). The lobar extent/severity of the following HRCT abnormalities were scored semiquantitatively [15, 16]: (1) the extent of bronchiectasis (0, no bronchiectasis; 1, bronchiectasis in 1 or part of 1 bronchopulmonary segment; 2, bronchiectasis in >1 bronchopulmonary segment; and 3, generalized bronchiectasis); (2) the severity of bronchiectasis (0, no airway dilatation; 0.5, trivial dilatation compared with the transverse diameter of the homologous pulmonary artery; 1, 100%–200% of that diameter; and 2, more than 200%–300% of that diameter); (3) the severity of bronchial wall thickening (0, no discernible wall thickening; 0.5, trivial wall thickening; 1, <50% of the transverse diameter of the homologous pulmonary artery; 2, 50%–100% of the transverse diameter of the accompanying pulmonary artery); (4) the extent of centrilobular (small airway); (5) large-airway mucus plugging (both similarly scored as follows: 0, no plugging; 1, plugging in 1 or part of 1 bronchopulmonary segment; 2, plugging in >1 bronchopulmonary segment; and 3, generalized plugging in all bronchopulmonary segments); (6) the severity of lobar volume loss (0, no volume loss; 1, mild volume loss; and 2, marked volume loss). Thus, the maximal expressible score for each of the extent of bronchiectasis, small-airway plugging, and large-airway plugging was 18, and the maximal score for severity of bronchiectasis, bronchial wall thickening and volume loss was 12.

After semiquantitative evaluation, the overall extent of abnormal lung in each lobe (to the nearest 5%) and the proportional extents of the following HRCT patterns (totaling 100%) were quantified: consolidation, a reticular pattern, ground-glass opacification, decreased attenuation (as a component of the mosaic attenuation pattern), emphysema, and pulmonary air cysts and nodules, with or without cavitation. Discrepant observations for the presence or absence of the radiological patterns and their distribution were resolved by consensus review. The threshold for consensus was a difference in proportional extent of abnormalities of >20%.

### Statistical Analysis

Data were extracted from paper-based questionnaires by optical character recognition (Cardiff Teleform Intelligent Character, version 10.7), and analyzed using Stata software (version 12; (=StataCorp). Data were expressed as means with SDs or medians with interquartile range (IQR) or range, as appropriate. A height-for-age and weight-for age *z* score of ≤2, based on World Health Organization reference standards, was used to define stunting and wasting, respectively [17]. For chest radiography, the score for each pattern was recorded as a mean value of both observers’ scores. Similarly, for the 6 semiquantitatively scored HRCT abnormalities, the lobar scores for each observer were first summed, and the average for the 2 observers was then calculated. Because the maximal expressible scores for the semiquantitative variables differed, the semiquantitative data were linearly transformed into a percentage scale, wherein 0 represented normal appearances, and 100 the maximal extent of abnormality, to homogenize the scores [18]. Thus, for example, a patient with a bronchiectasis extent score of 6 and a bronchiectasis severity score of 6 would have his or her scores expressed as 6/18 × 100 = 33.3 and 6/12 × 100 = 50.0, respectively.

For the total extent of abnormality and the proportional extent of the 8 CT patterns assessed, the post-consensus lobar scores for each observer were first summed, divided by 6 to obtain a mean lobar score, and the average lobar score for the 2 observers was then calculated. Observer agreement data for quantifying the extent/severity of chest radiographic and HRCT patterns was assessed using the weighted quadratic Cohen’s kappa value (κ_ω_) for ordinal variables or the single-determination (SD) [19] for continuous variables as appropriate. Agreement was categorized as poor (κ_ω_, 0 to ≤0.20), fair (0.20 to ≤0.40), moderate (0.40 to ≤0.60), good (0.60 to ≤0.80), or excellent (0.80 to ≤1.00) [19]. The association between common HRCT patterns (>10% prevalence) and between HRCT patterns and FEV_1_ was examined using Spearman rank correlation coefficients (*r*_s_).

## RESULTS

Of the 202 participants recruited into the study, 193 had chest radiographic data available; the median age of participants was 11.2 years (IQR, 9.0–12.8) years, and 46% were female. The median duration of ART was 4.8 years (IQR, 2.6–6.4) and 79% had an HIV load <400 copies/mL. The chest radiograph was assessed as abnormal by the respiratory physician in 44% of participants and by the thoracic radiologists in 33% (64 of 193). The most common principal abnormality was ring/tramline opacities (seen in 28% of participants), followed by consolidation (seen in 7%). Other patterns were uncommon (Table 1).

**Table 1.** Prevalence and Extent of Chest Radiographic Abnormalities

Abnormality	Participants, No. (%)	
	Prevalence of Abnormality	With >25% Extent of Abnormality^a^
Principle patterns		
Rings or tramlines	55 (28)	54 (98)
Consolidation	14 (7)	10 (71)
Ground-glass pattern	10 (5)	7 (70)
Reticulation	1 (0.5)	0 (0)
Noncavitating nodules	3 (2)	3 (100)
Cavitating nodules	0	…
Mass	0	…
Nonprincipal patterns		
Volume loss	10 (5)	NA
Hyperexpansion	4 (2)	NA
Lymph nodes	8 (4)	NA
Paucity of markings	3 (2)	NA

Abbreviation: NA, not applicable.

^a^Given as a percentage of those exhibiting the abnormality.

**Figure 1.**
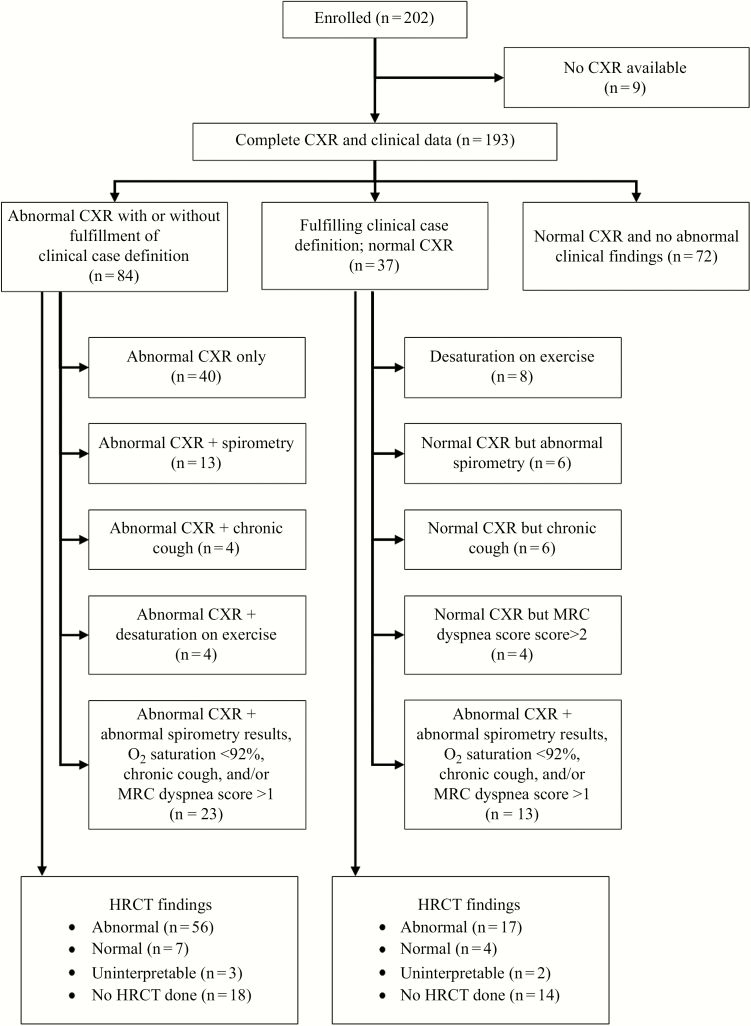
Flow chart of study participants. Abbreviations: CXR, chest radiography; HRCT, high-resolution computed tomography; MRC, Medical Research Council; O_2_, oxygen.

Overall, 121 participants (63%) were eligible for HRCT scanning owing either to abnormal clinical findings or to an abnormal chest radiograph (Figure 1). Of these, 89 (74%) underwent HRCT scanning (the remainder not attending for their appointment), of whom 5 were excluded because of image degradation by breathing artifact. Among those eligible for HRCT, there were no differences between those who did and those who did not undergo HRCT in age or sex, duration of ART, proportion with viral suppression, or proportion with respiratory symptoms and signs. Clinical findings are summarized in Table 2 but are discussed in detail elsewhere [10].

**Table 2.** Characteristics of Study Participants Undergoing Chest Radiography and High-Resolution Computed Tomography

Characteristic	Participants, No. (%)^a^	
	Undergoing Chest Radiography (n = 193)	Undergoing HRCT (n = 84)
Female sex	89 (46)	44 (52)
Age, median (IQR), y	11.2 (9.0–12.8)	11.6 (9.6–13.6)
Age at diagnosis, median (IQR), y	4.9 (2.7–7.5)	5.5 (3.3–8.5)
Duration of ART, median (IQR), y	4.8 (2.6–6.4)	4.7 (2.7–6.6)
CD4 cell count, median (IQR), cells/µL	720 (473–947)	686 (447–887)
HIV load <400 copies/mL	150 (79)^b^	64 (77)^c^
Any respiratory symptom	51 (26)	32 (38)
MRC dyspnea score >1	22 (11)	15 (18)
Daily cough for >1 mo	30 (16)	21 (25)
Daily cough for >1 mo with sputum	18 (12)	13 (15)
Wheezing	9 (5)	4 (5)
Household member who is a smoker	42 (22)	23 (27)
Height-for age *z* score ≤2	69 (36)	32 (38)
Weight-for age *z* score ≤2	18 (9)	9 (11)
Known history of asthma	7 (4)	2 (2)
History of *Pneumocystis jirovecii*	6 (3)	1 (1)
Previously treated for tuberculosis	74 (38)	34 (40)
Tachypnea (respiratory rate >30/min at rest for age 6–12 y or >24/min for age 13–16 y)	12 (6)	6 (7)
Abnormal SpO_2_ (resting hypoxia <92% or desaturation >5% with exercise)	23 (12)	15 (18)
Abnormal results of spirometry^d^	40 (24)	27 (37)
Obstructive pattern at spirometry	7 (5)^e^	5 (10)^f^
Chest radiograph identified as abnormal by respiratory physician	84 (43)	…

Abbreviations: ART, antiretroviral therapy; HIV, human immunodeficiency virus; IQR, interquartile range; MRC, Medical Research Council; SpO_2_, oxygen saturation.

^a^Data represent No. (%) of participants unless otherwise specified.

^b^Viral load data were missing in 3 participants.

^c^Viral load data were missing in 2 participants.

^d^Spirometric results were of acceptable quality for 127 participants in the chest radiography group and 73 in the HRCT subgroup.

^e^Spirometric data available in 136 individuals.

^f^Spirometric data available in 51 individuals.

**Figure 2.**
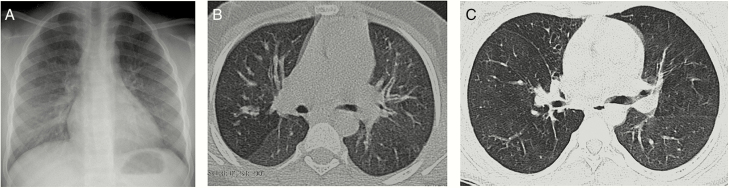
*A,* Chest radiograph of a participant scored as normal independently by both radiologists. *B,* High-resolution computed tomographic (HRCT) scan of the same patient, through the midzone demonstrating a striking mosaic pattern with areas of decreased attenuation adjacent to regions of normal lung density. *C,* HRCT scan of another patient, again showing regions of decreased attenuation as part of the mosaic pattern with cylindrical bronchiectasis in the middle lobe.

HRCT abnormalities were present in 70 of 84 participants (83%); the chest radiograph was normal in 39 participants (56%) with HRCT abnormalities. The most common HRCT abnormality was bronchial wall thickening, observed in 82% of HRCT scans, but this was of trivial severity in the majority (Tables 3 and 4). Decreased attenuation, as part of a mosaic attenuation pattern, was present in 43% of scans; 35 of the 36 scans with decreased attenuation had >5% extent of the pattern (Figure 2). Bronchiectasis, lobar volume loss, and large- or small-airway plugging were seen in 33%, 29%, and 26% respectively, and emphysema in 7%. Consolidation (2%), reticulation (1%), ground-glass opacification (1%), and pulmonary air cysts (1%) were uncommon HRCT findings.

**Table 3.** Prevalence and Extent/Severity of High-Resolution Computed Tomographic Scan Abnormalities

HRCT Pattern	Participants, No. (%)			
	Prevalence of Pattern	Relative Proportion of Severity^a^		
		0 < Score < 25	25 < Score < 50	Score ≥50
Bronchiectasis extent	28 (33)	19 (68)	8 (29)	1 (4)
Bronchiectasis severity	NA	17 (61)	9 (32)	2 (7)
Bronchial wall thickening	69 (82)	51 (74)	16 (23)	2 (3)
Small-airway plugging	12 (14)	12 (100)	0 (0)	0 (0)
Large-airway plugging	10 (12)	7 (70)	3 (30)	0 (0)
Lobar volume loss	24 (29)	22 (92)	1 (4)	1 (4)

Abbreviation: NA, not applicable.

^a^Percentage of total with pattern.

**Table 4.** Proportional Extent of High-Resolution Computed Tomographic Scan Patterns

HRCT Pattern	Participants, No. (%)	
	Prevalence of Pattern	Pattern of >5% HRCT Extent^a^
Consolidation	2 (2)	2 (100)
Reticulation	1 (1)	1 (100)
Ground-glass opacity	1 (1)	1 (100)
Decreased attenuation	36 (43)	35 (97)
Emphysema	6 (7)	6 (100)
Air cysts	1 (1)	1 (100)
Cavitating nodules	0 (0)	0 (0)
Noncavitating nodules	0 (0)	0 (0)

^a^Given as a percentage of those exhibiting the HRCT pattern.

The extent of decreased attenuation, as a component of the mosaic attenuation pattern, was strongly correlated with the extent of bronchiectasis (*r*_s_ = 0.68; *P* < .001) and the severity of bronchial wall thickening (*r*_s_ = 0.63; *P* < .001). There were weaker associations between the extent of decreased attenuation and plugging of large (*r*_s_ = 0.40; *P* < .001) or small (*r*_s_ = 0.38; *P* < .001) airways. The severity of bronchial wall thickening was also strongly correlated with the extent of bronchiectasis (*r*_s_ = 0.61; *P* < .001) and with plugging of large (*r*_s_ = 0.49; *P* < .001) or small (*r*_s_ = 0.50; *P* < .01) airways. Decreased attenuation and the extent of bronchiectasis were strongly correlated with FEV_1_ (*r*_s_ = −0.52; *P* < .001 and *r*_s_ = −0.50; *P* < .001 respectively) (Table 5).

**Table 5.** Correlation Between High-Resolution Computed Tomographic Patterns and Lung Function (Indices of Airflow Limitation)

HRCT abnormality	Association With Lung Function, Spearman Rank Correlation Coefficient, *r*_s_ (*P* Value)			
	FEV_1_	FVC	FEF_25%–75%_	FEV_1_/ FVC
Bronchiectasis extent	−0.50 (<.001)	−0.36 (.002)	−0.44 (<.001)	−0.26 (.03)
Bronchiectasis severity	−0.49 (<.001)	−0.36 (.002)	−0.44 (<.001)	−0.26 (.03)
Bronchial wall thickening	−0.43 (<.001)	−0.34 (.003)	−0.34 (.003)	NS
Small-airway plugging	−0.40 (<.001)	−0.33 (.005)	−0.37 (.01)	NS
Large-airway plugging	−0.42 (<.001)	−0.38 (.001)	−0.36 (.002)	NS
Volume loss	−0.38 (.001)	−0.34 (.003)	NS	NS
Decreased attenuation	−0.52 (<.001)	−0.42 (<.001)	−0.42 (<.001)	NS

Abbreviations: FEF_25%–75%_, forced expiratory flow, mid-expiratory phase; FEV_1_, forced expiratory volume in first second of expiration; FVC, forced vital capacity; NS, not significant.

Observer variability for quantifying the extent of abnormal lung on chest radiographs was moderate (κ_ω_ = 0.56) (Tables 6 and 7). Observer agreement for semiquantitative HRCT abnormalities was moderate to good, and the single-determination SD for the 7 continuous HRCT variables ranged from 5.8% for consolidation to 31.0% for decreased attenuation.

**Table 6.** Interobserver Agreement for Evaluation of Radiological Patterns at Chest Radiography and High-Resolution Computed Tomography: Continuous Variables

Pattern	sdSD, %
Chest radiography	
Consolidation	19.7
Ground-glass opacities	16.4
Reticulation	1.0
Noncavitating nodules	11.5
Cavitating nodules	5.0
Ring/tramline opacities	28.9
Masses	0.0
HRCT	
Overall extent	12.2
Consolidation	5.8
Ground-glass opacity	6.2
Reticulation	5.6
Decreased attenuation	31.0
Emphysema	18.2
Cysts	7.1

Abbreviations: NA, not applicable; sdSD, single-determination standard deviation.

**Table 7.** Interobserver Agreement for Evaluation of Radiological Patterns at Chest Radiograph and High-Resolution Computed Tomographic: Ordinal Variables

Pattern	κ_ω_
Chest radiography	
Overall extent	0.56
Paucity of vessels	NA
Hilar/mediastinal nodes	NA
Volume loss	NA
HRCT	
Bronchiectasis extent	0.66
Bronchiectasis severity	0.68
Bronchial wall thickening	0.60
Small-airway plugging	0.59
Large-airway plugging	0.81
Volume loss	0.64

Abbreviations: NA, not applicable.

## DISCUSSION

Our study shows that there is a high prevalence of lung disease in HIV-infected children receiving ART. The most common abnormality on plain chest radiographs were ring and tramline opacities, suggestive of airway inflammation. However, HRCT is the imaging modality of choice for the investigation of small- and large-airway disease. The principal HRCT abnormality were regions of decreased attenuation admixed with lung of normal density, the “mosaic attenuation pattern.” Because of its lower contrast resolution, the mosaic pattern is not seen on chest radiographs. By contrast, HRCT is exquisitely sensitive to any alteration in density, which, for the lung parenchyma, is strictly governed by the amount of air. Any change in the *relative* proportion of air, whether this is physiological (as is seen during normal breathing) or pathological (as might occur with any infiltrative disease; with impaired ventilation caused by small-airway disease and specifically, obliterative bronchiolitis [OB]; or with altered perfusion [eg, chronic thromboembolic disease]), will increase or decrease parenchymal attenuation [20].

In our patients, there was no clinical evidence of an infiltrative disease, such as infection of pulmonary edema. Moreover, the strong association between the extent of decreased attenuation and indices of airflow obstruction (FEV_1_ and forced expiratory flow, midexpiratory phase), without reversibility, makes chronic thromboembolic disease highly unlikely and instead supports OB as the likely cause of chronic lung disease in HIV-infected children. Importantly, there was a strong correlation between the extent of decreased attenuation and the extent of bronchiectasis, again strongly supporting airway-based pathology.

Histopathological [21] and CT studies [22] have shown that bronchiectasis and OB frequently coexist. Moreover, studies in which HRCT patterns have been correlated with functional indices have shown that OB may precede the onset of bronchiectasis, as opposed to the suppuration and dilatation of the large airways leading to OB [22, 23]. Notably, our finding that OB is the likely major abnormality accounting for chronic lung disease contrasts sharply with findings from the pre-ART era, when lymphoid interstitial pneumonitis was the most common cause of chronic lung disease in children [24]. Lymphoid interstitial pneumonitis responds well to ART and corticosteroid treatment and is now uncommon [25].

It is important to appreciate that OB is not a diagnosis per se but a pathological lesion. Obliteration of the small airways (defined arbitrarily as those with an internal diameter <2 mm) is a common incidental pathological finding [26, 27]. The underlying mechanism is inflammation, which then causes partial or complete obliteration [28]. OB can occur as a sequel to diverse “insults,” including viral lower respiratory tract infections, connective tissue diseases, and exposure to toxic fumes, or as a manifestation of chronic graft-vs-host disease in recipients of heart-lung transplants [28]. A biopsy might be considered the reference standard for diagnosis, but the lesions of OB are patchily distributed and without meticulous dissection; (compounded by issues of sampling error), the diagnosis is easily overlooked [29]. Furthermore, there is a high risk associated with performing an open (surgical) biopsy in children with already compromised respiratory function, particularly in low-resource settings.

Chest radiography is widely available, of low cost, and has a relatively low radiation dose, but it is an insensitive tool to identify OB: in our study, 56% of individuals with an abnormal HRCT scan had a normal chest radiograph. In addition, the 2-dimensional nature of the chest radiograph (leading to anatomic superimposition), and poor contrast resolution inevitably cause interpretative difficulties [30]. Therefore, HRCT coupled with lung function testing are probably the best noninvasive tests for a diagnosis of OB.

However, owing to concerns about radiation dose, there is reluctance to perform HRCT studies in children. In addition, HRCT is not routinely available in resource-limited settings, which explains why this condition has not to date been identified in busy African HIV clinics. In the absence of alternative diagnoses, individuals with chronic symptoms are often treated presumptively for tuberculosis, a disease that is common in settings with high HIV prevalence.

An interesting finding in our study was the presence of emphysema (albeit of limited extent) in 7% of study participants. CT studies in a total of 1681 HIV-infected adults from 3 series [31–33], have shown a prevalence of emphysema ranging from 19% to 53%. This large variation is, in part, almost certainly caused by different criteria for defining “significant” emphysema, and, even after controlling for smoking status, HIV was identified as an independent risk factor for emphysema [31].

There is a high reported prevalence of chronic lung disease, particularly airflow obstruction, among HIV-infected individuals, but mainly in high-income settings and among adults, where smoking and substance use is a major confounder. Importantly, none of the participants in our cohort had ever smoked, and biomass fuel use is relatively low in our setting, compared with other urban and periurban settings in sub-Saharan Africa [10, 34, 35]. In addition, the prevalence of respiratory symptoms and poor lung function was substantially higher than in uninfected children of the same age group and socioeconomic status, as reported by Rylance et al [10]. The pathogenesis of chronic lung disease in HIV-infected children is not well understood but may be a long-term consequence of repeated respiratory tract infections or a direct consequence of HIV infection itself.

One strength of our study was that participants were not selected on the basis of symptoms. Subjects underwent HRCT scanning based on a predefined clinical case definition informed by previous studies that have shown a similar clinical picture in this age group [7]. In addition, radiological data was complemented by detailed clinical and lung function data. Furthermore, all radiological studies were scored independently by 2 experienced thoracic radiologists. A limitation of the study was that HRCT was performed only in participants who had clinical features of chronic lung disease or were assessed to have an abnormal chest radiograph, and some children with subclinical disease may have been missed, given the low sensitivity of chest radiography. HRCT scans were not available in 31% of participants. However, this was owing to participants not attending their scan appointments, and there was no difference in demographic or clinical features between those who did and did not attend. We were unable to measure lung diffusion capacity. There was some interobserver variation in scoring HRCT scans and chest radiographs, but this is within the range expected and both radiologists reached consensus on discrepant observations. It was not feasible to confirm our findings histologically. The study is cross-sectional, and therefore no causality can be attributed.

To our knowledge, ours is the first large prospective study of HRCT findings in HIV-infected older children and adolescents, and it demonstrated airway disease to be the major cause of chronic lung disease in this age group, in contrast to findings of lymphoid interstitial pneumonitis in the pre-ART era. Studies to elucidate the natural history and etiology are urgently needed so that therapeutic strategies can be developed.

## Notes

***Author contributions.*** J. M., K. K., and R. A. F. designed the study and undertook the literature search. S. R. D. and A. N. undertook quantification of chest radiographic and HRCT abnormalities. A. N. and J. R. analyzed the data (with input from S. R. D. and R. A. F.). G. M. and E. M. coordinated the study. H. M., K. N., and G. M. provided clinical care. H. M. and K. N. contributed to the study design and study logistics. S. R. D. wrote the first draft of the manuscript. S. R. D. and R. A. F. had full access to all the data in the study and final responsibility for the decision to submit for publication. All authors contributed to writing the report and have approved the final draft.

***Disclaimer.*** The funding sources had no role in the writing of the manuscript or in the decision to submit for publication.

***Financial support.*** This work was supported by the Nina Ireland Program for Lung Health (J. M.) and the Wellcome Trust through an Intermediate Fellowship (grant 095878/Z/11Z to R. A. F.).

***Potential conflicts of interest.*** All authors: No reported conflicts of interest. All authors have submitted the ICMJE Form for Disclosure of Potential Conflicts of Interest. Conflicts that the editors consider relevant to the content of the manuscript have been disclosed.
